# Retraction Notice to: MicroRNA-4500 Inhibits Migration, Invasion, and Angiogenesis of Breast Cancer Cells via RRM2-Dependent MAPK Signaling Pathway

**DOI:** 10.1016/j.omtn.2025.102658

**Published:** 2025-08-07

**Authors:** Shaoying Li, Huifen Mai, Yefeng Zhu, Guofeng Li, Jing Sun, Guisen Li, Bichan Liang, Shaojun Chen

## Main text

(Molecular Therapy: Nucleic Acids *21*, 278–289; September 2020)

*Molecular Therapy Nucleic Acids* is retracting this article at the request of the authors and the editor-in-chief. Concerns regarding image duplication were brought to the attention of the editorial office. These concerns are described in a Pubpeer thread (https://pubpeer.com/publications/9D80E15B721AAB810B1C92391E30C9) in which readers drew attention to similarities between the figures in this article and the figures in two articles published in *Cancer Cell International* (Wang et al., 2019, Cancer Cell Int. *19*, 114, https://doi.org/10.1186/s12935-019-0808-z; Jiang et al., 2019, Cancer Cell Int. *19*, 271, https://doi.org/10.1186/s12935-019-0977-9) by unrelated authors. Image analysis performed by the editorial office revealed evidence of image duplication in Figures 4A, 7A, and S4A of this article. This reuse of data without appropriate attribution represents a severe abuse of the scientific publishing system. The original notice of retraction was undeliverable to co-author Jing Sun; however, a formal retraction request, signed by all authors, was received by the editorial office after the notice was sent.

